# Neuronal correlates of social decision making are influenced by social value orientation—an fMRI study

**DOI:** 10.3389/fnbeh.2015.00040

**Published:** 2015-02-24

**Authors:** Katarina Kuss, Armin Falk, Peter Trautner, Christian Montag, Bernd Weber, Klaus Fliessbach

**Affiliations:** ^1^Center for Economics and Neuroscience, University of BonnBonn, Germany; ^2^Department of Psychiatry, University Hospital BonnBonn, Germany; ^3^Life and Brain Center, Department of NeuroCognition, University Hospital BonnBonn, Germany; ^4^Department of Psychology, University of UlmUlm, Germany; ^5^Department of Epileptology, University Hospital BonnBonn, Germany; ^6^Clinical Research, German Center for Neurodegenerative Diseases (DZNE)Bonn, Germany

**Keywords:** prosocial decision making, interindividual differences, SVO, egoistic default, valuation, cognitive control

## Abstract

Our decisions often have consequences for other people. Hence, self-interest and other-regarding motives are traded off in many daily-life situations. Interindividually, people differ in their tendency to behave prosocial. These differences are captured by the concept of social value orientation (SVO), which assumes stable, trait-like tendencies to act selfish or prosocial. This study investigates group differences in prosocial decision making and addresses the question of whether prosocial individuals act intuitively and selfish individuals instead need to control egoistic impulses to behave prosocially. We address this question via the interpretation of neuronal and behavioral indicators. In the present fMRI-study participants were grouped into prosocial- and selfish participants. They made decisions in multiple modified Dictator-Games (DG) that addressed self- and other-regarding motives to a varying extent (self gain, non-costly social gain, mutual gain, costly social gain). Selfish participants reacted faster than prosocial participants in all conditions, except for decisions in the non-costly social condition, in which selfish participants displayed the longest decision times. In the total sample we found enhanced neural activity in the ventromedial prefrontal cortex (vmPFC) and dorsomedial prefrontal cortex (dmPFC/BA 9) during decisions that resulted in non-costly social benefits. These areas have been implicated in cognitive control processes and deliberative value integration. Decisively, these effects were stronger in the group of selfish individuals. We believe that selfish individuals require more explicit and deliberative processing during prosocial decisions. Our results are compatible with the assumption that prosocial decisions in prosocials are more intuitive, whereas they demand more active reflection in selfish individuals.

## Introduction

Prosocial behavior, i.e., behavior which benefits other individuals is a relative unique human ability. There is an ongoing scientific debate to what extent prosocial behavior is based on intuition or on conscious reasoning (Fehr and Camerer, 2007). Recent studies suggest that this might strongly depend on personality traits, and that individuals with a tendency to act prosocial (“prosocials”) do so intuitively, while individuals who have a tendency to act selfish, sometimes rely on their ability to deliberatively control their selfish tendencies in order to act prosocial (Bogaert et al., 2008; Declerck et al., 2013). Neurocognitive research has recently begun to identify brain processes which are related to prosocial behavior and they have found that prosocial decisions in persons with a prosocial personality trait are associated with activity in subcortical brain regions that have been implied in automated, intuitive processing (Haruno and Frith, 2010; Haruno et al., 2014). In the present fMRI study we wanted to investigate brain processes in selfish individuals which are given the opportunity to act prosocially without own costs. Under the assumption that these individuals have a weaker default tendency to act prosocial we hypothesized that they would need extra cognitive resources to do so manifesting in longer reaction times and stronger activity in brain areas that are associated with deliberative decision making such as the prefrontal cortex.

Standard models of human decisions making assume that individuals are intuitively self-interested and primarily maximize their own gain (Camerer, 2003). Therefore, individuals have to suppress egoistic impulses in order to act prosocially (Knoch et al., 2006). Compatible with this view, egoistic choices are fast (Piovesan and Wengström, 2009) and presuambly automated. On the contrary, a study by Rand and colleagues suggests that individuals are intuitively prosocial and cooperative (Rand et al., 2012). They found that faster decisions result in higher monetary contributions to a public good than slow decisions.

Studies that take interindividual differences into account, suggest that these conflicting views might be resolved by controlling for personality traits. The concept “Social Value Orientation” (SVO) (Van Lange, 1999) considers two kinds of stable (trait-like) preferences for resource allocation: a prosocial value orientation, which refers to the preference of maximizing the sum of resources between self and other, and a proself value orientation, which refers to the preference of maximizing individual resources. Cornelissen et al. (2011) studied the effects of social value orientation on prosocial behavior in a dictator-game under cognitive load during which prosocials transferred more money to the recipient compared to selfish participants. The authors concluded that SVO determines behavior when it is based on automatization, resulting prosocials to intutiveily act in a prosocial manner and selfish individuals to intuitively act in an egoistic manner.

Additional support for this view comes from neurocognitive studies that explore differences in brain acitivity between prosocial and selfish individuals (Van den Bos et al., 2009; Haruno and Frith, 2010; Emonds et al., 2011; Haruno et al., 2014). They found evidence that behavior and cognition of prosocials are characterized by intuition and automatization. Work by Haruno and colleagues demonstrated that prosocials rely more on automatic emotional processing when responding to inequitable monetary distributions between themselves and others. They found correlates in subcortical structures such as the Amygdala and the Nucleus Accumbens (Haruno and Frith, 2010; Haruno et al., 2014). This finding was complemented by another study that provided evidence for a higher reward value of prosocial decisions in prosocial-oriented individuals: Van den Bos found higher activity in the Striatum of prosocials compared to proselfs when reciprocating in a Trust game (Van den Bos et al., 2009). Similarly Emonds et al. (2011) interpreted higher activity in the lateral orbitofrontal cortex in prosocials during decision making in a Prisoner's Dilemma as neural indicators of intuitive, internalized moral consideration. Neural indicators for deliberative strategies and control of egoistic impulses in proselfs are reported in the same study (Emonds et al., 2011). They found higher activity in the DLPFC in proselfs for decision making in the Prisoner's Dilemma. The DLPFC has been considered to be an important brain region for working memory, because it is activated when humans are under cognitive load. Therefore higher activity in this brain area might reflect high cognitive effort to fight automatic egoistic impulses.

**Figure 1:**
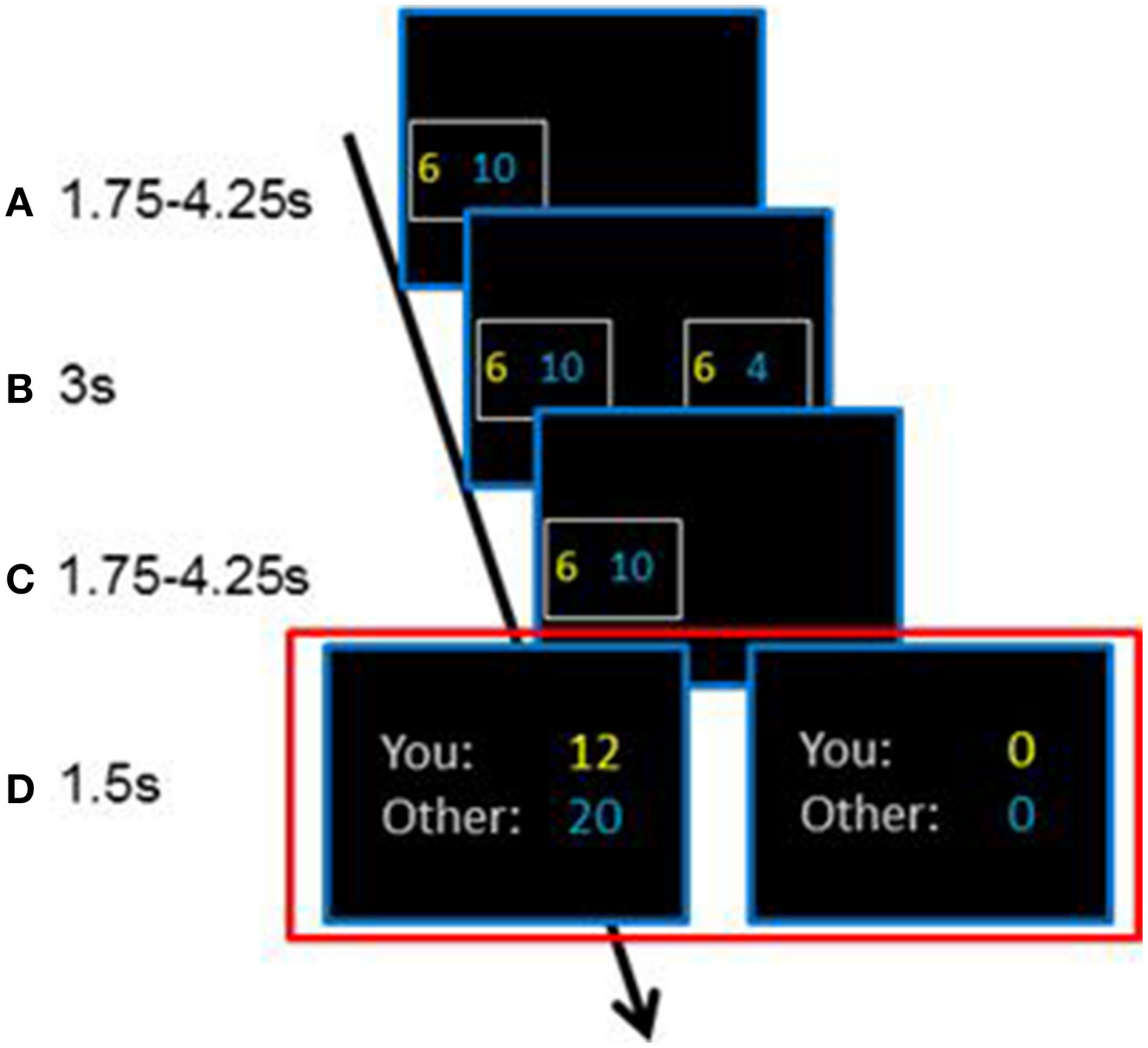
**Chronology of a trial in the paradigm**. After the appearance of the first alternative **(A)**, consisting of a payoff for the decision-maker in yellow and the receiver in blue, a second alternative appeared and the subjects chose one alternative **(B)**. The chosen alternative was presented **(C)** and the chosen outcomes were either doubled or set to zero with a 50% chance **(D)**. The chronology was the same in each of the 160 trials.

Our fMRI study aimed at extending those findings to decision-making in a non-interactive paradigm. We are interested in the decision process that underlies the construct of social value orientation and thus implemented a binary choice task that resembles the structure of the triple dominance measure of SVO (Van Lange, 1999). Participants choose between two alternatives, each alternative consisting of a payoff for themselves and another participant (example see Figure 1). Our paradigm conceptually resembles a dictator-game (Engel, 2011): decisions affect payoffs of the decision maker and of another participant (the recipient), without the recipient having influence on the payoff distribution. This excludes the possible influence of strategic motives and motives to punish unfair behavior which are effective in interactive paradigms such as the Ultimatum game (Camerer, 2003).

Our paradigm allowed to study the valuation and integration of self-interest and prosocial motives and the relative contribution of different brain structures to prosocial decisions. We expected prosocials to act intuitively in a prosocial manner, and selfish individuals to act intuitively in an egoistic manner. The main question was how selfish individuals would react in situations in which they could act prosocially without minimizing their personal benefit. Either they don't act prosocially (at most at chance level), or they act prosocial and need additional cognitive resources to do so. To answer this question we designed situations (non-costly social condition) which focus on the payoff maximization of the other person (by holding the own payoff constant and varying the other person's payoff, e.g., choosing between 4/10 and 4/6). Since these decisions do not lead to monetary losses for the decision-maker, we expected a high rate of prosocial decisions. Decisions in the costly social condition entail a conflict between self-interest and other-regarding motives. In this situation subjects can choose to forgo a monetary advantage for themselves in order to allocate more money to the recipient (e.g., favoring 4€ for themselves and 16€ for the recipient over 6€ for both). In those situations we expected prosocials participants to behaviourally display their value for prosocial outcomes by choosing the prosocial alternative more often compared to selfish individuals.

Our study is able to shed light on the neural correlates underlying the decision process of the SVO construct and the relative contribution of brain areas involved in more automated processing (such as the Nucleus Accumbens and the Amygdala) and brain areas involved in higher order reflective processing (such as the lateral and medial prefrontal cortex) (Satpute and Lieberman, 2006). We wanted to contribute to the question, whether individuals that value prosociality (i.e., prosocials) primarily act intuitively, and whether selfish individuals need to control egoistic impulses to behave prosocially. We expected prosocials to show stronger neural correlates of reward and valuation in subcortical structures of the brain (NAcc, Amygdala), and we expected selfish indidivuals to show stronger engagement of prefrontal areas during prosocial decisions.

## Methods

### Participants

Prior to the fMRI experiment, each of our 40 subjects (22 female, mean age = 30.03y, SD = 8.7y) was classified as proself (*n* = 20) or prosocial (*n* = 20) based on the Social Value Orientation decomposed measure (Van Lange, 1999), which was filled out online. The SVO decomposed measure consists of 9 items. Each item contains three outcome distributions between oneself and an anonymous other, in this case another participant of the online-questionnaire (points to self/points to other). The three outcome distributions of each item correspond to a prosocial (e.g., 500/500), individualistic (e.g., 600/200) or competitive orientation (e.g., 500/0). Participants were classified as prosocial when they made at least 6 consistent prosocial choices, and classified as proself when they made at least 6 consistent individualistic choices. Competitive choices were too few to be classified. Participants were classified before the fMRI experiment to achieve an equal distribution of proself- and prosocial-oriented participants. Four subjects (all proselfs) had to be excluded from fMRI-analysis due to excessive head movement. Throughout the manuscript we use the term selfish to refer to proself individuals as defined in the SVO construct. Subjects were native German speakers, right handed and had no history of psychiatric or neurological disorders. Informed written consent was obtained from all subjects. The study was approved by the Ethics committee of the University of Bonn.

### Experimental procedure and paradigm

In each of the 160 trials of the fMRI experiment, subjects chose one of two alternatives, each consisting of a payoff for themselves and for another participant (Figure 1). Subjects were informed that the payoff of one randomly chosen trial would be implemented after the experiment (actual payoff). No deception was used: the selection of the implemented trial was random and the actual payoffs were transferred to the subjects (decision maker and another participant as the receiver) and subjects were guaranteed anonymity of their decisions.

Subjects were invited in groups of two and took part in the fMRI-experiment one after another. The choices the subjects made affected each other; each subject had the role of a dictator and of a receiver, thus the paradigm corresponds to a role-reversal dictator game. The randomly chosen actual payoff at the end of each experiment thus determined a payoff for the decision-maker (dictator) and a payoff for the other subject of the dyad as the receiver. The actual payoff for each participant henceforth consisted of the sum of two randomly chosen decisions: one at the end of their own experiment, the other at the end of the other participant's experiment. The subjects additionally received a show-up fee of 20 Euro. The subjects didn't know each other in advance. They briefly recognized one another before the first subject entered the scanner in order to demonstrate the receiver to actually exist. The two participants had no further contact and didn't get to know the decisions of each other. We decided to implement minimal prior contact between the subjects in order to increase ecological validity. Additional several studies demonstrated a positive influence of prior contact and of perceived similarity on cooperative behavior and its neuronal correlates (Boone et al., 2008, 2010; Mobbs et al., 2009).

The decision-process consisted of three time-points (see Figure 1). After the appearance of the first alternative showing a payoff for the decision-maker and the receiver, a second alternative appeared and the subjects chose one alternative. After the choice, we implemented a Reward-Prediction-Error (RPE) by either doubling the chosen outcomes or setting them to zero with a 50% chance. This allows us to test for neuronal correlates before choice (during appearance of first alternative), during choice, and after choice (RPE) (for a more detailed description of this procedure see Supplementary Material).

Subjects' and receivers' payoffs varied independently among 4, 6, 10, 16, and 20 Euros, and payoff alternatives were randomly chosen from all possible unique combinations. This led to four qualitatively distinguishable decision situations: “pure self-interest” (PSI), “non-costly social” (NCS), “efficiency” (E) and “costly social” (CS) situations (The generation of decision situations is explained in detail in the Supplementary Material). In the costly social situations, subjects could choose to forgo monetary advantages in order to allocate more money to the receiver (e.g., favoring 6€ for themselves and 16€ for the receiver over 10€ for both). In this situation, subjects had to trade material self-interest with altruistic preferences. The other three situations do not entail a conflict between different motives because one alternative is unequivocally advantageous with respect to self-interest motives (PSI), efficiency (NCS), or both motives (E). The non-conflicting nature of the NCS-condition was created by keeping the subject's payoff constant in the two alternatives and only varying the receiver's outcome (See Table 1: 6/10 vs. 6/4). In this situation the subjects can choose the alternative with the higher outcome for the other participant without affecting the own outcome. The PSI condition was constructed in an equivalent manner by keeping the receiver's payoff constant and varying the subject's payoff (e.g., 10/6 vs. 4/6). In the efficient condition one alternative consisted of higher payoffs for decision-maker and receiver (16/10 vs. 10/6). Subjects were presented with 40 decisions of each condition in random order, resulting in 160 decisions in total. The experimental paradigm was adopted from our previous study (Kuss et al., 2013).

**Table 1:** **The four decision situations and their underlying payoff-structures including percentages of trials in which subjects chose the left alternative in each condition separately for prosocials and proselfs (selfish participants)**.

**Decision situation**	**Percentage of trials averaged across subjects (mean ± SD)**		
	**Prosocials**	**Proselfs**	***t*-value (p)**
Pure self-interest (PSI) e.g., 10/6 4/6	94.1% (± 12.92%)	95% (± 12.11%)	−0.22 (0.829)
Efficiency (E) e.g., 16/10 4/6	95.5% (± 8.96%)	95% (± 12.53%)	0.14 (0.886)
Non-costly social (NCS) e.g., 6/10 6/4	90.3% (± 16.25%)	92.1% (± 12.7%)	−0.36 (0.724)
Costly social (CS) e.g., 4/10 10/6	19.6% (± 16.32%)	6.9% (± 10.94%)	2.79 (0.008)

In order to characterize the neuronal correlates of social decision-making, the analysis of fMRI-data concentrates on the following comparison (time-point of choice, see Figure 1B): the contrast between social decisions in the NCS-condition with self-interested choices in the PSI-condition (NCS > PSI). This contrast is of special interest, because conditions are formally equivalent (no conflict of motives, 1 payoff per alternative is the same) and conditions differ only in the decision's consequence with NCS-choices affecting the receiver's payoff and PSI-choices affecting the decision-maker's payoff. The events of costly social decisions (costly-social condition) were too rare to be considered in the fMRI-analysis.

### Technical details

Scanning was performed on a 1.5T Avanto scanner (Siemens, Erlangen, Germany) using standard scanning parameters for the acquisition of 31 axial EPI slices with a TR of 2.5s (for details see Supplementary Material). The experiment was presented by Presentation® software version 14.9 (Neurobehavioral Systems, Albana, Canada) via video goggles (Nordic Neuro Lab, Norway) and subjects gave their answer by button presses on MRI-suited response grips (Nordic Neuro Lab).

### fMRI analysis

We included three events in the first level general linear model (GLM): onset of the appearance of the first alternative including parametric modulators representing the subject's payoff and the receiver's payoff (event 1), different onset-regressors depending on the decision situations (event 2), onset of RPE-induction including two parametric modulators representing the RPE of the subject's and the receiver's payoff (event 3). Regarding event 2, the following decision-types were modeled in the GLM: In the PSI condition trials where subjects chose the self-interest alternative (PSI+), in the NCS-condition trials where subjects choose the prosocial alternative (NCS+), in the E-condition trials where subjects chose the efficient alternative (E+), in the CS-condition trials where subjects chose the prosocial alternative (CS+), and trials where subjects chose the self-interest alternative (CS−). All decisions, except prosocial decision in the costly social condition (CS+), were made with a certain frequency to be considered as a reliable regressor in the GLM (at least 20 decisions per condition).

Importantly, the parametric regressor for the others's payoff in event 1 and event 3 were entered after the subject's payoff regressors and regressors were orthogonalized in ascending order. This means that in case of shared variance between these regressors, all commonly explained BOLD variance was attributed to the subject's regressors, yielding an independent and conservative estimate for the effect of the receiver's payoff regressors.

In order to test our hypotheses we contrasted the following decision types to describe the neuronal correlates of social decision making during event 2. We contrasted social decisions in NCS- and E-condition with self-interested decision in the PSI-condition (NCS > PSI, E > PSI). For this decision-related activity, we build differential t-contrasts on first level. Prosocial decisions in the CS-condition (CS+) were too rare to have reliable parameter estimates. We subjected the regressors of the parametric modulators of event 1 and event 3 to one-sample *t*-tests. We tested all contrasts in the whole sample (*n* = 36) and for group differences (prosocial versus selfish participants).

The voxel-level threshold was set to 0.005 (uncorrected). We applied a whole-brain cluster-level family-wise error (FWE) correction for multiple comparisons with a cluster-p-value of 0.05. Additionally we applied a small-volume correction for a-priori defined Regions of Interest (ROI).

### ROI definition

We defined regions known to be involved in valuation and reward processing as regions of interest, namely the Nucleus Accumbens (NAcc) and the medial orbitofrontal cortex (mOFC). Additionally, we were interested in the subgenual ACC, as this region was shown to be involved in reward-processing in a social context (Moll et al., 2006; Kuss et al., 2013). We derived anatomical masks of these regions from the Harvard-Oxford cortical and subcortical structural atlases (http://www.cma.mgh.harvard.edu), applying a probability of 0.5. Further we used the AAL as implemented in SPM 8 to derive masks for Caudate and Putamen as approximation to a mask for the Ventral Striatum.

## Results

### Behavioral results

Decisions in conditions without conflict (non-costly social and efficiency) demonstrate that individuals make social decisions that profit the other person at no cost to self-interest (Table 1). In pure self-interest (PSI), non-costly social (NCS) and efficiency (E) situations, all subjects consistently chose the advantageous alternative (>92% of all trials over all subjects): the choices in PSI were advantageous with respect to self-interest, in NCS advantageous with respect to social gain, and in E advantageous with respect to both. Table 1 shows the choice behavior in the four conditions separately for prosocial and selfish individuals.

In costly social situations which were characterized by a conflict between prosocial and self-interest motives, prosocial participants were more often willing to forgo own monetary advantages in order to distribute more money to the receiver. Prosocials more often chose the prosocial alternative in this condition when compared to selfish participants. For the other conditions there was no significant difference in choice behavior between the groups (see Table 1).

**Figure 2:**
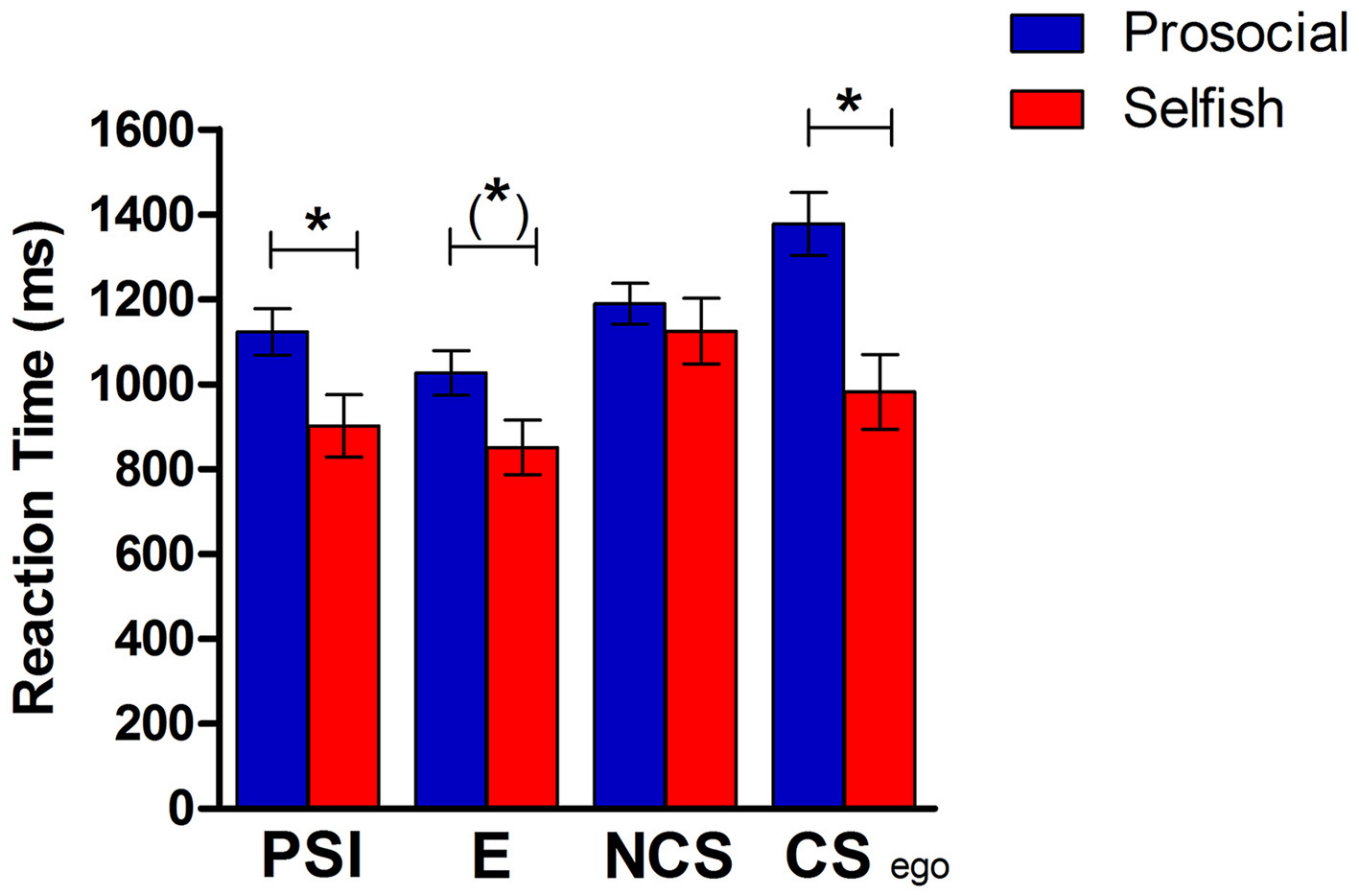
**Reaction-times in the four conditions separately for the group of prosocial and selfish participants**. PSI, choosing the self-interest alternative in pure self-interest condition; E, choosing efficient alternative in efficiency condition; NCS, choosing social alternative in the non-costly social condition; CS ego, choosing the self-interest alternative in costly-social condition. ^*^p < 0.05; (^*^)*p* < 0.1.

In the analysis of reaction-times we considered decisions that were also included in the GLM of the fMRI-analysis. Reaction-time refers to the event where subjects choose one alternative (Figure 1B) and is defined as time from appearance of the second alternative (Figure 1B) until button press (choosing one alternative). There was a significant difference in reaction-times between conditions [*F*_(3, 102)_ = 41.13, *p* < 0.001] and an interaction-effect [condition X group: *F*_(3, 102)_ = 14.59, *p* < 0.001]. Reaction-times are shown in Figure 2, separately for prosocials and selfish participants. Prosocials took longer to decide in every experimental condition compared to selfish individuals. The group-differences were significant in PSI-condition [*t*_(34)_ = 2.21, *p* = 0.034] and for egoistic decisions in CS-condition [*t*_(34)_ = 3.29, *p* = 0.002]. The group-difference reaches a trend for choices in the E-condition [*t*_(34)_ = 1.74, *p* = 0.09], while there was no significant difference observed in the NCS-condition [*t*_(34)_ = 0.52, *p* = 0.609]. The pattern of reaction-time-differences is remarkable: Prosocials took longest for egoistic choices in the costly-social condition, whereas selfish participants took longest for social choices in the non-costly-social condition.

### fMRI-results

The aim of the study was to describe differences in neuronal correlates of social-decision making between selfish- and prosocial-oriented subjects. After reporting results in the whole sample (*n* = 36), we present the group-difference between prosocial (*n* = 20) and selfish participants (*n* = 16) for the contrast of main interest (NCS > PSI).

**Figure 3:**
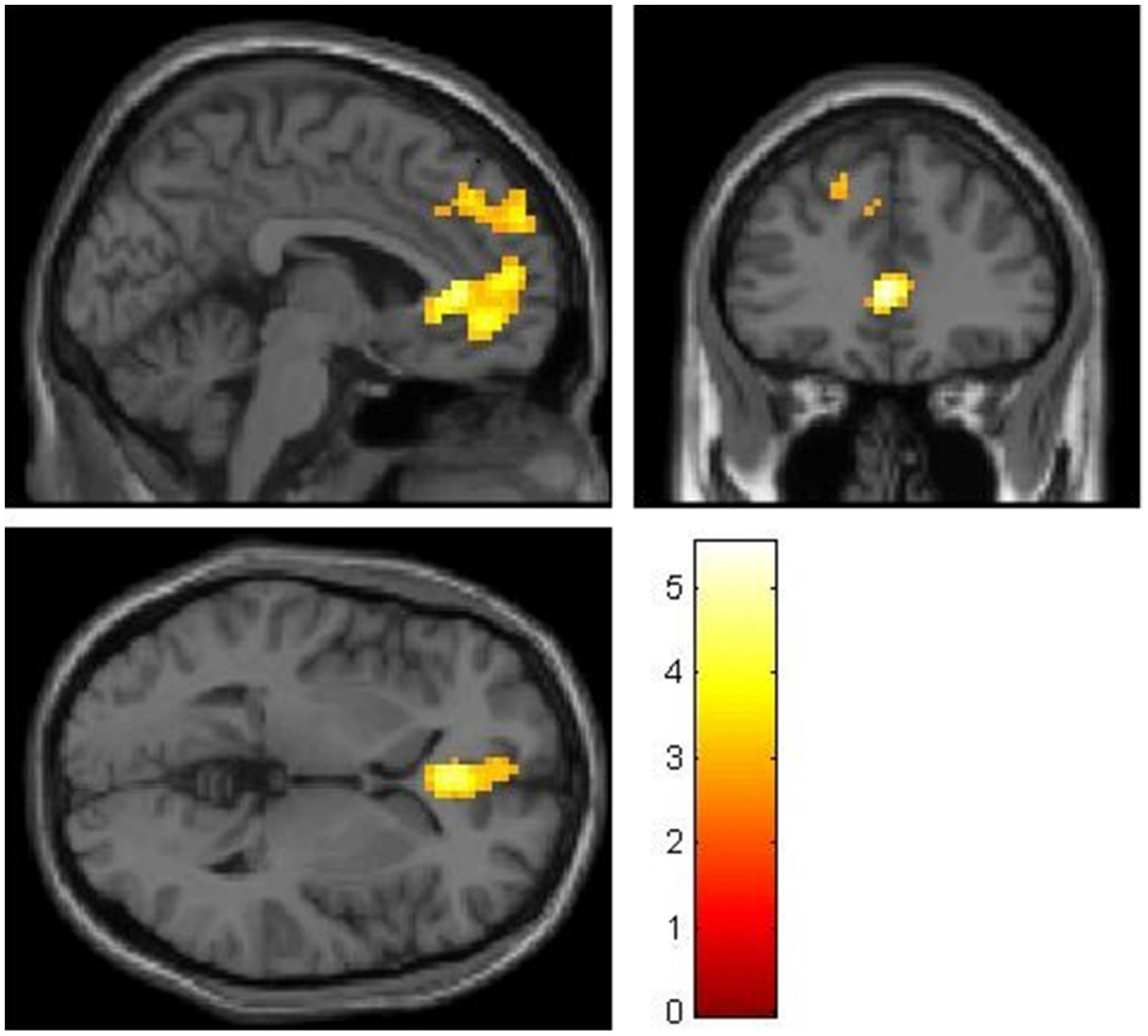
**Stronger BOLD-signal for non-costly social choices compared to pure-self interested choices (NCS > PSI) in vmPFC and dmPFC in the whole sample (*n* = 36)**. MNI: *X* = -3, *Y* = 35, *Z* = 2, thresholded at *t* > 2.73, corresponding to *p* < 0.005.

Non-costly social decisions (NCS) compared to self-interested choices (PSI) were associated with activations in two clusters located in medial frontal regions. One cluster was located in ventromedial areas, including the medial orbitofrontal cortex (from now on: ventromedial prefrontal cortex, vmPFC). The other cluster is more dorsal, located in BA9 (from now on: dorsomedial prefrontal cortex, dmPFC). Those two clusters survive correction for multiple comparison on a whole brain level: Non-costly social decisions (NCS) were associated with a stronger BOLD-Signal in the vmPFC [NCS > PSI: MNI-coordinates of peak voxel: *X* = 0, *Y* = 35, *Z* = 4, *t* = 5.53, *k* = 340, pFWE(whole brain cluster level)<0.05] and in the dmPFC [NCS > PSI: MNI-coordinates of peak voxel: *X* = −9, *Y* = 56, *Z* = 34, *t* = 4.15, *k* = 204, pFWE(whole brain cluster level)<0.05], as shown in Figure 3.

For the opposite contrasts (PSI > NCS), there was no activation in reward-related areas that survived correction for multiple comparisons.

**Figure 4:**
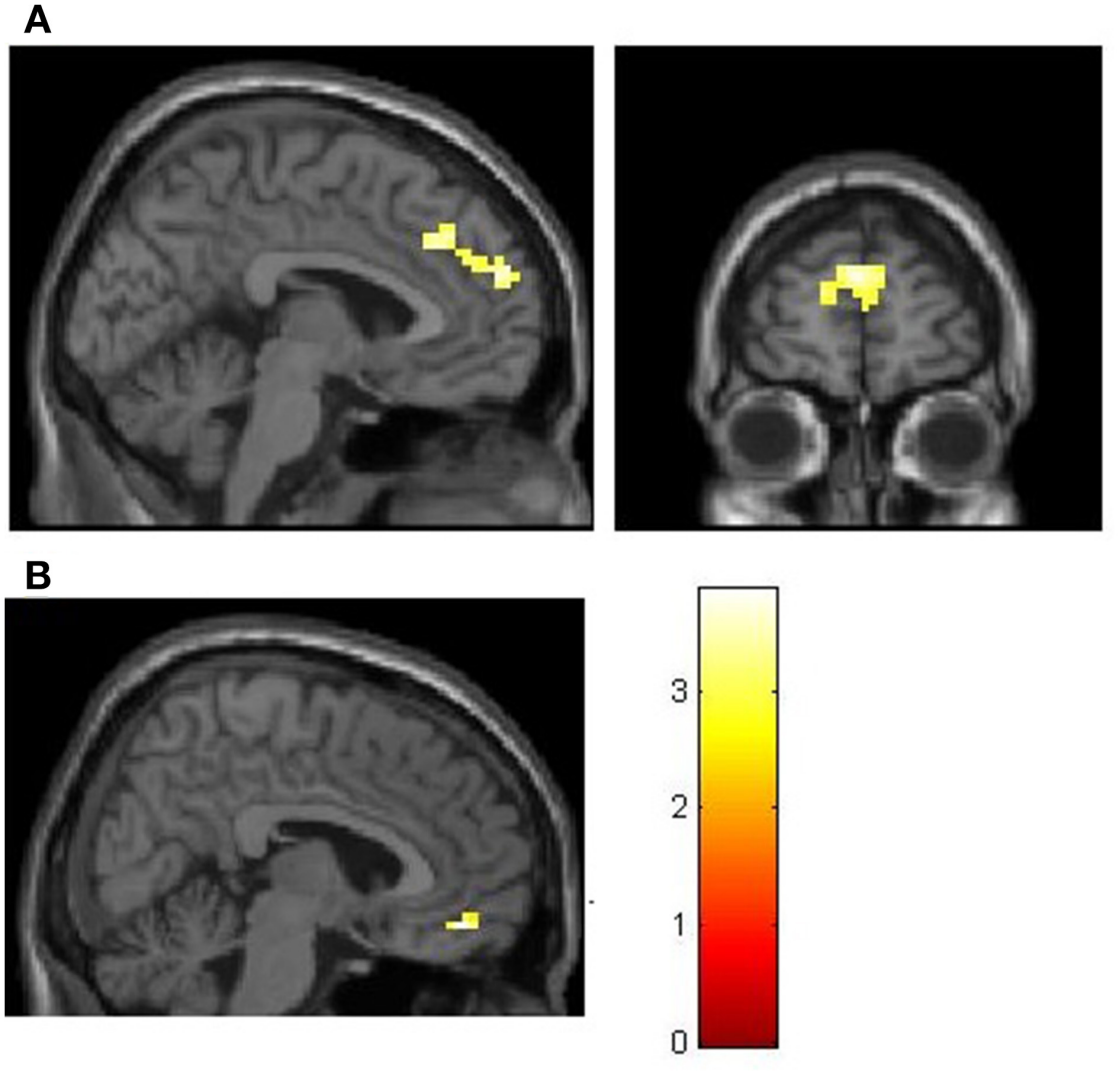
**Stronger BOLD-signal in the group of selfish participants for social compared to self-interested choices (NCS > PSI) in the dmPFC (A) and mOFC (B)**. **(A)** MNI: *X* = -3, *Z* = 22. **(B)** MNI: *X* = 6. Both are thresholded at *t* > 2.73, corresponding to *p* < 0.005.

These correlates of social decision making (NCS > PSI) were tested for group-differences between prosocial and selfish participants. There was no stronger activity in the group of prosocials (prosocials > proselfs). Instead there was a stronger BOLD-signal in the group of proselfs for social compared to self-interested choices (NCS > PSI) in the dmPFC [MNI-coordinates of peak voxel: *X* = 0, *Y* = 32, *Z* = 34, *t* = 3.86, *k* = 172, pFWE(whole brain cluster level)<0.05] and in the mOFC [MNI-coordinates of peak voxel: *X* = 6, *Y* = 47, *Z* = −14, *t* = 5.01, pFWE(small-volume corrected)<0.05] when compared with the group of prosocials (proselfs > prosocials, see Figure 4).

Further results of prosocial decision making in the efficient condition and results of the RPE-event are reported in the Supplementary Material.

## Discussion

This study demonstrates inter-individual differences in behavioral and neuronal correlates of prosocial preferences between prosocial and selfish individuals (Van Lange, 1999). We found that prosocials more frequently choose to allocate money to the receiver despite own losses in the costly social condition upon comparison to selfish pariticipants. This finding is more or less circular because the SVO measure used to discriminate the groups actually uses a very similar task. In fact, this result implies a validation for the fact that our fMRI paradigm resembles the definition of the SVO concept.

For the purpose of this paper, we focused on a condition where subjects could make prosocial choices (administering more money to the receiver) without any consequences for the own payoff (non-costly social condition). In this condition the large majority of subjects chose the prosocial alternative and selfish and prosocial participants did not differ in this issue. Crucially we investigated reaction time differences and differences in BOLD signal during those choices in order to elucidate the underlying cognitive processes and their differences in the two groups.

Reaction times were significantly longer in prosocial than in selfish individuals in all conditions except for the non-costly social (NCS) condition yielding a significant group x condition interaction. A similar interaction was found for BOLD activity in the vmPFC and dmPFC, where the contrast between NCS and PSI (pure self-interest) was larger for selfish than for prosocial individuals. The NCS condition is the only condition where self-beneficial choices cannot be made. Selfish individuals are therefore required to overcome their default of primarily considering their own outcome.

### Group differences in reaction times as indicators of an egoistic default in selfish individuals

One possibility to experimentally disentangle automated and controlled processes is to manipulate cognitive load (Satpute and Lieberman, 2006). Cornelissen et al. (2011) studied the effects of social value orientation on prosocial behavior in a dictator-game under cognitive load during which prosocials transferred more money to the recipient compared to selfish participants. The authors concluded that chronically accessible values are automatically transferred into behavior.

In our paradigm, group differences in reaction-times allow for conclusions on the automaticity of decision-processes in prosocial and selfish participants. Selfish participants react faster in all conditions compared to prosocials. Those differences were significant, except for non-costly social decisions. We assume that selfish participants automatically chose the options with the higher payoff for themselves. This option is easy to determine in all conditions, except for the non-costly social condition (here the own payoff is constant in both alternatives). In this condition, selfish-oriented participants are prompted to look at the payoff for the receiver and take it into account. Prosocial-oriented participants instead consider the receiver's payoff in all decisions, thus taking longer to decide in general.

This is clearly the case in the costly social condition: Here prosocial participants take very long to decide, which reflects the conflict between self-interest and prosocial motives in this condition. Selfish participants instead are quite fast in this decision, presumably because they use the heuristic of choosing the alternative with the highest payoff for themselves. While the fast reactions of selfish participants can be regarded as an indicator of intuition in following an egoistic decision default, the slow responses of prosocials can be seen as an indicator of deliberation in the costly social condition where self-interest and prosocial motives are in conflict (Rubinstein, 2007; Kahneman, 2011). In the underlying value-based decision process, prosocials obviously place a positive value on the outcome of the other person, whereas selfish indiviuals primarily value their own outcomes.

### Neural correlates of overriding this egoistic default in areas of value computation, cognitive control and social cognition are more pronounced in selfish individuals

Our results demonstrate activity during social choices in brain areas that are associated with on the one hand (1) subjective (reward-) value (mOFC, vmPFC) and on the other hand (2) theory of mind, executive function and cognitive control (dmPFC).

Besides its reward-related function, the vmPFC is also associated with the integration of costs and benefits (De Quervain et al., 2004; Basten et al., 2010) and with choosing alternatives with high subjective value (Rangel and Hare, 2010; Bartra et al., 2013). Decisions in our paradigm require the integration of the value of the personal payoff and the value of the receiver's payoff into one subjective value. The vmPFC activity in our paradigm was observed for non-costly social decisions. Non-costly social choices in our paradigm induce selfish individuals to consider the payoff of another person, and thus induces a valuation-process that adds on the selfish default of considering mainly their own payoff. The vmPFC activity was stronger in the group of selfish participants implying higher demand on value computation in selfish individuals, compared to the more intuitive prosocial decisions of prosocials.

In a similar vein, higher activity in the dmPFC in selfish individuals during non-costly social choices can be seen as a correlate of reflection and displays the need of cognitive resources (and thus less automaticity) in individuals who primarily use an egoistic decision default.

The dmPFC is associated with cognitive control and controlled forms of social cognition as opposed to automatic forms of social cognition (Satpute and Lieberman, 2006; Lieberman, 2007). Different cognitive processes have been reported to be associated with dmPFC activity, especially for tasks that require cognitive control and computational load (e.g., Elliott and Dolan (1998) for higher cognitive demands during hypothesis testing; Ferstl and von Cramon (2002) during coherency processing of speech; Berthoz et al. (2002) during processing of norm violations; Decety et al. (2004) during competition). Those results demonstrate the dmPFC' s role for the maintenance of non-automated cognitive processes and hint at a more general and domain-independent function (Ferstl and von Cramon, 2002). In this vein, the dmPFC activity during non-costly social choices can be regarded as an indicator of a reflective cognitive process, which is stronger in selfish-oriented participants.

The dmPFC (BA9) is also associated with the processing of socially relevant stimuli, theory of mind and mentalizing (Gallagher and Frith, 2003; Saxe, 2006). Stronger activation during social decisions in selfish participants could also reflect a higher demand of theory of mind- and executive-functions in this group. In a similar vein, Krueger et al. (2007) report group-differences in BA 9 during the course of a trust-game: the group of participants that did not experience reciprocity in the first half of the experiment had stronger activity in BA9 compared to participants who experienced their trust being reciprocated. Participants that did not experience reciprocity rely more on mentalizing processes in order to predict the behavior of the other. Participants that did however experience reciprocity during the game, trust more automatically and thus demand fewer mentalizing processes. (Krueger et al., 2007). This parallels our group-differences during social choices in BA9, because we assume prosocials to use social cognition more automatically compared to selfish individuals.

Neural indicators for a reward value of prosocial decisions were observed as well. Activity in the mOFC and vmPFC during social choices imply that decisions, which profit another person at no cost to the decision maker, carry an intrinsic reward-value. Our results confirm results of previous studies that reported reward-related activity during social choices in interactive cooperation paradigms (Decety et al., 2004; Rilling et al., 2004; Elliott et al., 2006; Emonds et al., 2011), as well as in non-interactive paradigms (Moll et al., 2006; Mobbs et al., 2009; Tricomi et al., 2010; Zaki and Mitchell, 2011; Fareri et al., 2012).

To conclude, our results hint at the need for controlled processes in prosocial decision making. Additionally prosocial decisions at no cost to self-interest seem to have an intrinsic reward-value. Furthermore our results demonstrate interindividual differences in neuronal correlates of prosocial decision making. These decisions seem to recruit the need to overcome the default of maximizing self-interest in selfish individuals, which is accompanied by activity in areas associated with controlled forms of social cognition such as the dmPFC (Lieberman, 2007) and areas associated with the integration of values such as the vmPFC (Rangel and Hare, 2010). The results allow a more detailed view on prosocial decision making that takes interindividual differences into account: it does not seem to be a question of deliberation versus intuition *per se*. Instead, selfish-oriented individuals apply reflection and need cognitive resources to overcome self-interest and act prosocially, whereas prosocial-oriented individuals seem to rely more on intuitive processes.

## Author contributions

KK, KF, BW, CM, AF designed the experiments. KK, CM, PT programmed the experiment. KK and CM conducted the experiment. KK, PT, KF analyzed data. BW, AF, KF reviewed and supervised data analysis. KK, KF, CM, BW, AF discussed and interpreted the data. KK, KF, CM, BW wrote the manuscript.

### Conflict of interest statement

The authors declare that the research was conducted in the absence of any commercial or financial relationships that could be construed as a potential conflict of interest.

## References

[B1] Bartra O. McGuire J. T. Kable J. W. (2013). The valuation system: a coordinate-based meta-analysis of BOLD fMRI experiments examining neural correlates of subjective value. Neuroimage 76, 412–427. 10.1016/j.neuroimage.2013.02.063 23507394 PMC3756836

[B2] Basten U. Biele G. Heekeren H. R. Fiebach C. J. (2010). How the brain integrates costs and benefits during decision making. Proc. Natl. Acad. Sci. U.S.A. 107, 21767–21772. 10.1073/pnas.0908104107 21118983 PMC3003102

[B3] Berthoz S. Armony J. L. Blair R. J. R. Dolan R. J. (2002). An fMRI study of intentional and unintentional (embarrassing) violations of social norms. Brain 125, 1696–1708. 10.1093/brain/awf190 12135962

[B4] Bogaert S. Boone C. Declerck C. (2008). Social value orientation and cooperation in social dilemmas: a review and conceptual model. Br. J. Soc. Psychol. 47, 453–480. 10.1348/014466607X244970 17915044

[B5] Boone C. Declerck C. Kiyonari T. (2010). Inducing cooperative behavior among proselfs versus prosocials: the moderating role of incentives and trust. J. Confl. Resolut. 54, 799–824 10.1177/00220027I0372329

[B6] Boone C. Declerck C. Suetens S. (2008). Subtle social cues, explicit incentives, and cooperation in social dilemmas. Evol. Hum. Behav. 29, 179–188 10.1177/00220027I0372329

[B7] Camerer C. F. (2003). Behavioral Game Theory: Experiments in Strategic Interaction. Princeton, NJ:Princeton University Press.

[B8] Cornelissen G. Dewitte S. Warlop L. (2011). Are social value orientations expressed automatically? Decision making in the dictator game. Pers. Soc. Psychol. Bull. 37, 1080–1090. 10.1177/0146167211405996 21518808

[B9] Decety J. Jackson P. L. Sommerville J. A. Chaminade T. Meltzoff A. N. (2004). The neural bases of cooperation and competition: an fMRI investigation. Neuroimage 23, 744–751. 10.1016/j.neuroimage.2004.05.025 15488424 PMC3640982

[B10] Declerck C. H. Boone C. Emonds G. (2013). When do people cooperate? The neuroeconomics of prosocial decision making. Brain Cogn. 81, 95–117. 10.1016/j.bandc.2012.09.009 23174433

[B11] De Quervain D. J. -F. Fischbacher U. Treyer V. Schellhammer M. Schnyder U. Buck A.. (2004). The neural basis of altruistic punishment. Science 305, 1254–1258. 10.1126/science.1100735 15333831

[B12] Elliott R. Dolan R. J. (1998). Activation of different anterior cingulate foci in association with hypothesis testing and response selection. Neuroimage 8, 17–29. 10.1006/nimg.1998.0344 9698572

[B13] Elliott R. Völlm B. Drury A. McKie S. Richardson P. Deakin J. F. W. (2006). Co-operation with another player in a financially rewarded guessing game activates regions implicated in theory of mind. Soc. Neurosci. 1, 385–395. 10.1080/17470910601041358 18633801

[B14] Emonds G. Declerck C. H. Boone C. Vandervliet E. J. M. Parizel P. M. (2011). Comparing the neural basis of decision making in social dilemmas of people with different social value orientations, a fMRI study. J. Neurosci. Psychol. Econ. 4, 11–24 10.1037/a0020151

[B15] Engel C. (2011). Dictator games: a meta study. Exp. Econ. 14, 583–610. 10.1007/s10683-011-9283-725222635

[B16] Fareri D. S. Niznikiewicz M. A. Lee V. K. Delgado M. R. (2012). Social network modulation of reward-related signals. J. Neurosci. 32, 9045–9052. 10.1523/JNEUROSCI.0610-12.2012 22745503 PMC3412567

[B17] Fehr E. Camerer C. F. (2007). Social neuroeconomics: the neural circuitry of social preferences. Trends Cogn. Sci. 11, 419–427. 10.1016/j.tics.2007.09.002 17913566

[B18] Ferstl E. C. von Cramon D. Y. (2002). What does the frontomedian cortex contribute to language processing: coherence or theory of mind? Neuroimage 17, 1599–1612. 10.1006/nimg.2002.1247 12414298

[B19] Gallagher H. L. Frith C. D. (2003). Functional imaging of “theory of mind.” Trends Cogn. Sci. 7, 77–83. 10.1016/S1364-6613(02)00025-6 12584026

[B20] Haruno M. Frith C. D. (2010). Activity in the amygdala elicited by unfair divisions predicts social value orientation. Nat. Neurosci. 13, 160–161. 10.1038/nn.2468 20023652 PMC3145100

[B21] Haruno M. Kimura M. Frith C. D. (2014). Activity in the nucleus accumbens and amygdala underlies individual differences in prosocial and individualistic economic choices. J. Cogn. Neurosci. 26, 1861–1870. 10.1162/jocn_a_00589 24564471

[B22] Kahneman D. (2011). Thinking, Fast and Slow. New York, NY: Farrar, Straus and Giroux.

[B23] Knoch D. Pascual-Leone A. Meyer K. Treyer V. Fehr E. (2006). Diminishing reciprocal fairness by disrupting the right prefrontal cortex. Science 314, 829–832. 10.1126/science.1129156 17023614

[B24] Krueger F. McCabe K. Moll J. Kriegeskorte N. Zahn R. Strenziok M.. (2007). Neural correlates of trust. Proc. Natl. Acad. Sci. U.S.A. 104, 20084–20089. 10.1073/pnas.0710103104 18056800 PMC2148426

[B25] Kuss K. Falk A. Trautner P. Elger C. E. Weber B. Fliessbach K. (2013). A reward prediction error for charitable donations reveals outcome orientation of donators. Soc. Cogn. Affect. Neurosci. 8, 216–223. 10.1093/scan/nsr088 22198972 PMC3575724

[B26] Lieberman M. D. (2007). Social cognitive neuroscience: a review of core processes. Annu. Rev. Psychol. 58, 259–289. 10.1146/annurev.psych.58.110405.085654 17002553

[B27] Mobbs D. Yu R. Meyer M. Passamonti L. Seymour B. Calder A. J.. (2009). A key role for similarity in vicarious reward. Science 324, 900. 10.1126/science.1170539 19443777 PMC2839480

[B28] Moll J. Krueger F. Zahn R. Pardini M. de Oliveira-Souza R. Grafman J. (2006). Human fronto-mesolimbic networks guide decisions about charitable donation. Proc. Natl. Acad. Sci. U.S.A. 103, 15623–15628. 10.1073/pnas.0604475103 17030808 PMC1622872

[B29] Piovesan M. Wengström E. (2009). Fast or fair? A study of response times. Econ. Lett. 105, 193–196 10.1016/j.rconlet.2009.07.017

[B30] Rand D. G. Greene J. D. Nowak M. A. (2012). Spontaneous giving and calculated greed. Nature 489, 427–430. 10.1038/nature11467 22996558

[B31] Rangel A. Hare T. (2010). Neural computations associated with goal-directed choice. Curr. Opin. Neurobiol. 20, 262–270. 10.1016/j.conb.2010.03.001 20338744

[B32] Rilling J. K. Sanfey A. G. Aronson J. A. Nystrom L. E. Cohen J. D. (2004). The neural correlates of theory of mind within interpersonal interactions. Neuroimage 22, 1694–1703. 10.1016/j.neuroimage.2004.04.015 15275925

[B33] Rubinstein A. (2007). Instinctive and cognitive reasoning: a study of response times. Econ. J. 117, 1243–1259 10.1111/j.1468-0297.2007.02081.x

[B34] Satpute A. B. Lieberman M. D. (2006). Integrating automatic and controlled processes into neurocognitive models of social cognition. Brain Res. 1079, 86–97. 10.1016/j.brainres.2006.01.005 16490183

[B35] Saxe R. (2006). Uniquely human social cognition. Curr. Opin. Neurobiol. 16, 235–239. 10.1016/j.conb.2006.03.001 16546372

[B36] Tricomi E. Rangel A. Camerer C. F. O'Doherty J. P. (2010). Neural evidence for inequality-averse social preferences. Nature 463, 1089–1091. 10.1038/nature08785 20182511

[B37] Van den Bos W. van Dijk E. Westenberg M. Rombouts S. A. Crone E. A. (2009). What motivates repayment? Neural correlates of reciprocity in the Trust Game. Soc. Cogn. Affect. Neurosci. 4, 294–304. 10.1093/scan/nsp009 19304843 PMC2728629

[B38] Van Lange P. A. M. (1999). The pursuit of joint outcomes and equality in outocomes: an integrative model of social value orientation. J. Pers. Soc. Psychol. 77, 337–349. 18316061

[B40] Zaki J. Mitchell J. P. (2011). Equitable decision making is associated with neural markers of intrinsic value. Proc. Natl. Acad. Sci. U.S.A. 108, 19761–19766. 10.1073/pnas.1112324108 22106300 PMC3241792

