# Supplementary material for: Neuronal correlates of social decision making are influenced by social value orientation—an fMRI study
Source: Front Behav Neurosci. 2015 Feb 24;9:40. doi: 10.3389/fnbeh.2015.00040 (PMC4338788; doi:10.3389/fnbeh.2015.00040)
Supplement: Supplementary file 1 [file Presentation1.PDF]

Supplementary Material for

Neuronal correlates of social decision making are influenced by social value orientation – an fMRI study

Katarina Kuss[1,4] , Armin Falk[1], Peter Trautner[2], Christian Montag[3], Bernd Weber[1,2,5], Klaus Fliessbach[1,4,6]

[1] Center for Economics and Neuroscience, University of Bonn, Bonn, Germany; [2] Life & Brain Center, Department of NeuroCognition, University Hospital of Bonn, Bonn, Germany; [3] Department of Psychology, University of Ulm, Ulm, Germany; [4] Department of Psychiatry, University Hospital Bonn, Bonn, Germany; [5] Department of Epileptology, University Hospital Bonn, Bonn, Germany; [6] German Center for Neurodegenerative Diseases (DZNE), Bonn, Germany

Methods

RPE model

The term reward prediction error (RPE) originates from studies of reinforcement learning. Here, a mismatch between an actual outcome and an expected outcome is thought to be the critical moment that drives adaptive learning (Schultz, 1997). More generally, RPEs arise when rewards occur in an unforeseeable manner. We induce uncertainty concerning the outcomes, by either doubling or setting the chosen outcomes to zero with a 50% probability. Having 160 decisions in the experiment, this results in a pool of 80 doubled chosen outcomes, and 80 zeroed chosen outcomes. Out of this pool, one chosen outcome was randomly drawn at the end of the experiment for the actual payoff.
In general the prediction error term can be defined as

$$RPE = R - EV$$

Where R is the reward magnitude of the actual outcome and EV is the expected value in a given situation. EV is given by gain probability times reward magnitude (Yacubian et al., 2006). We calculated the RPEs separately for the payoff of the subject and the receiver, with a fixed gain probability of 50%. In Figure 1 the subject choose 6 Euro for himself, 10 Euro for the other participant. Following we demonstrate the RPE-calculation for the own outcome. The expected value is 6 Euro (EV = 12 Euro * 0.5). In case of doubling the chosen outcomes (R = 12), the resulting RPE is positive (RPE = 12 – 6), in case of setting the chosen outcomes to zero the RPE is negative (RPE = 0-6).

The RPE were calculated on a trial by trail basis separately for the decision maker and the receiver, and were entered as parametric modulators in the first level GLM. For a similar approach on, see Kuss et al. (2013).

Payoff structure and decision types

We implemented 5 categories of subject´s and receiver´s payoff (4€, 6€, 10€, 16€, 20€) resulting in 25 possible combinations of the two payoffs into an alternative (A/B, represented in the rows and columns of Figure S1 respectively). Those 25 alternatives can be assembled into 625 combinations of the two alternatives represented in the cells of Supplementary Figure S1 (decisions-situations: A1/B1, A2/B2). Twenty-five of those are of no interest, since the two alternatives are identical (black cells of Supplementary Figure S1). Three-hundred unique decision-situations can be classified into one of the following four decision situation types (the remaining 300 decision-situations are identical, except for the order of alternative 1 and 2, and are referred to as mirrored situations; grey cells in Supplementary Figure S1):

1. Pure-self-interest situations (PSI, yellow cells in Supplementary Figure S1).
2. Non-costly-social situations (NCS, green cells in Supplementary Figure S1).
3. Efficiency situations (E, blue cells in Supplementary Figure S1).
4. Costly-social situations (CS, red cells in Supplementary Figure S1)

During the experiment, subjects were confronted with 160 trials randomly drawn out of the 600 decision-situations of interest, 40 out of each situation type. Half of the 160 decision-situations presented were drawn from the mirrored situations for each decision situation type, in order to balance the appearance of alternatives between left and right.

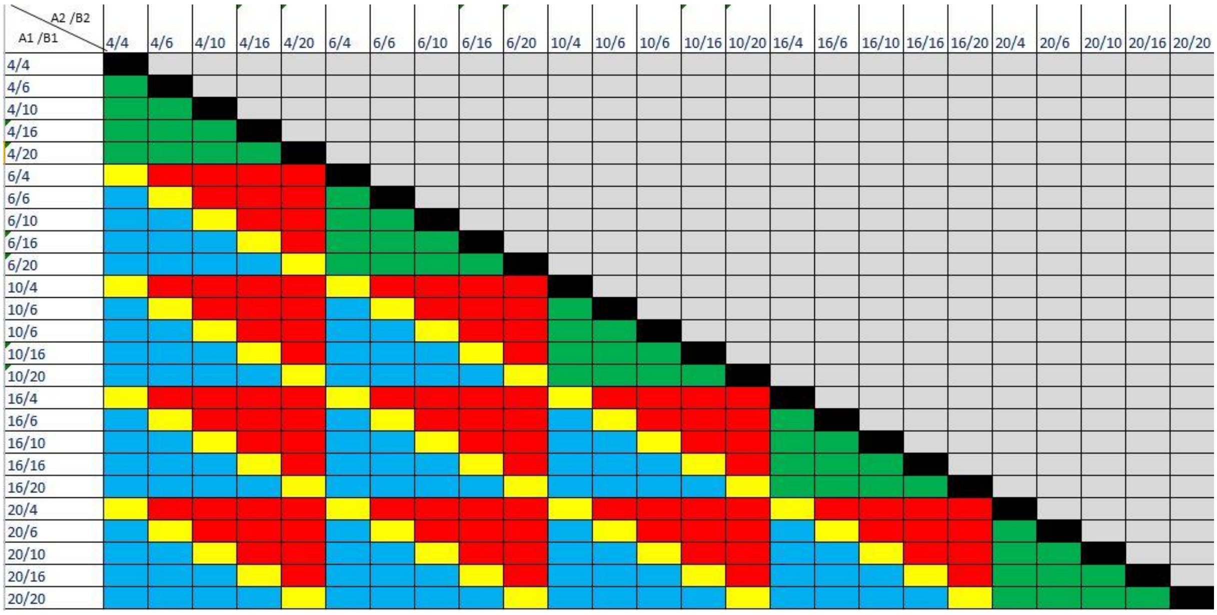

Figure S1. Payoff Structure and the classification into 4 decisions situation types
Notes: A: subject´s payoff, B: receiver's payoff; A1/B1: alternative 1; A2/B2: alternative 2; 50 yellow cells: Pure-self-interest; 50 green cells: Non-costly-social; 100 blue cells: Efficiency; 100 red cells: Costly-social; 300 grey cells: mirrored situations (corresponding decisions situations except for the order of alternative 1 and 2)

FMRI-Results

The contrast of efficient and self-interested choices reveals a stronger BOLD-signal in the mOFC (E > PSI: MNI-coordinates of peak voxel: X=3,Y=50,Z=-8, t=3.76, pFWE(small-volume corrected)<0.05), and Caudate (see Figure S2). The activation in Caudate does not survive correction for multiple comparisons within the anatomically defined ROI (E>PSI: MNI-coordinates of peak voxel: X=-6,Y=8,Z=1, t=3.59, pFWE(small-volume corrected) = 0.066). This activation can be considered a trend. Additionally the activation extends the anatomical mask (15 voxel around X=-6, Y=8, Z=1, 8 of those are in the mask; 17 voxel around X=6, Y=11, Z=4, 7 of those are in the mask).

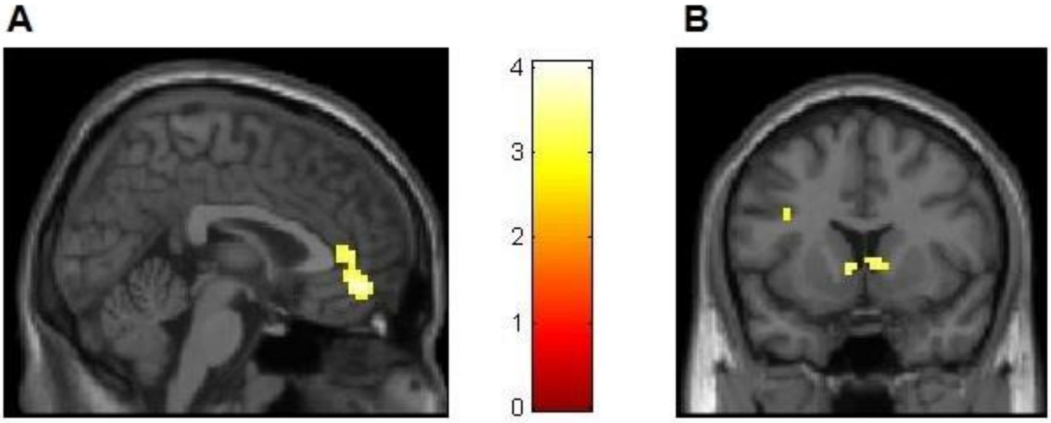

Figure S2. Stronger BOLD-signal for efficient choices compared to pure self-interested choices (E > PSI) in mOFC (Fig. 4A) and in Caudate (Fig. 4B) in the whole sample (n=36). Fig. 4A: MNI: X = -3. Fig. 4B: MNI: Z = 4. Both are thresholded at t>2.73, corresponding to p<0.005.

The comparison of efficient with self-interested choices (E > PSI) reveals stronger BOLD-signal in the group of proselfs compared to prosocials in mOFC (MNI-coordinates of peak voxel: X=3,Y=47,Z=-14, t=4.8, pFWE(small-volume corrected)<0.05), see Figure S3.

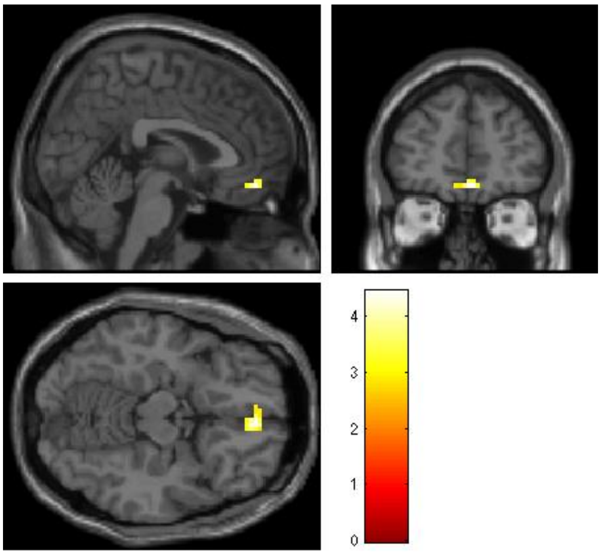

Figure S3. Stronger BOLD-signal in the group of proselfs for efficient compared to self-interested choices (E > PSI) in the mOFC. MNI**:** X = 3, Y = 47, Z = -14, thresholded at t>2.73, corresponding to p<0.005.

Besides neuronal activity during choice, we were interested in neuronal valuation-correlates post-choice. We implemented a Reward-Predicition-Error to test for outcome orientation independent of choice (Kuss et al., 2013).

We tested the RPE of the own monetary outcome and the RPE of the other person's outcome for a linear relationship with the BOLD-signal. There was a positive linear association of BOLD-signal with the RPE of the own monetary outcome in the NAcc (MNI-coordinates of peak voxel: X=9,Y=11,Z=-5, t=3.17, pFWE(small-volume corrected)<0.05). This effect is quite small (4 Voxel within the anatomically defined NAcc-ROI). As a robustness-check, we averaged the parameter estimates across the NAcc and confirmed the result: there was a significant positive linear relationship between BOLD and own RPE averaged across all voxels in the NAcc (t = 2.29, p = 0.013954). There was no such association in the other predefined ROI (mOFC, sgACC). Additionally, there was no group-difference between prosocials and proselfs. We tested for outcome orientation concerning the receiver's outcome. There was no positive linear relationship of BOLD with the RPE of the receiver that

survived correction for multiple comparisons in the predefined reward-related ROI. Additionally there was no significant positive relationship with the receiver's RPE, when averaging parameter estimates across the whole NAcc ($t = 0.79$, $p = 0.216$). There were no group-differences in this relationship.

Discussion of RPE results

We implemented a Reward-Prediction-Error in order to test for a neural reward value which is temporarily decoupled from the time-point of choice. We expected prosocial-oriented participants to have neural correlates in reward-related areas for the RPE of the social payoff. This would be a surrogate marker for the reward value prosocial participants attribute to payoffs affecting another person. Contrary to results of previous work (Kuss et al., 2013) we did not find neuronal correlates for social outcomes disentangled from choice (RPE-event). We found those outcome-oriented correlates for the own payoff in the NAcc, but couldn't find an equivalent signal for the receiver's payoff. The recipient in the study from 2013 was a charity organization and we were able to detect such a signal for the charity's payoff. The pattern of results in the present study hint at decision-related reward-activity during social choices and no outcome-oriented reward-activity for social outcomes disentangled from choice in a paradigm that affected another participant. Results of the charity-study showed reverse pattern of results (no decision-related, but outcome-related reward activity for social outcomes). We speculate that for decisions that affect another person, the act of decision is more relevant and potentially rewarding. For donation-decisions instead the neuronal processing of the outcome after the decision implies the relevance of the amount donated.

Literature

Kuss, K., Falk, A., Trautner, P., Elger, C. E., Weber, B., and Fliessbach, K. (2013). A reward prediction error for charitable donations reveals outcome orientation of donators. *Soc. Cogn. Affect. Neurosci.* 8, 216–23. doi:10.1093/scan/nsr088.

Schultz, W. (1997). A Neural Substrate of Prediction and Reward. *Science (80-.).* 275, 1593–1599. doi:10.1126/science.275.5306.1593.

Yacubian, J., Gläscher, J., Schroeder, K., Sommer, T., Braus, D. F., and Büchel, C. (2006). Dissociable systems for gain- and loss-related value predictions and errors of prediction in the human brain. *J. Neurosci.* 26, 9530–7. doi:10.1523/JNEUROSCI.2915-06.2006.
